# Atomic resolution map of the soluble amyloid beta assembly toxic surfaces†
†Electronic supplementary information (ESI) available: Methods, ^15^N-DEST profiles, and additional statistical analyses. See DOI: 10.1039/c9sc01331h


**DOI:** 10.1039/c9sc01331h

**Published:** 2019-05-21

**Authors:** Rashik Ahmed, Michael Akcan, Adree Khondker, Maikel C. Rheinstädter, José C. Bozelli, Richard M. Epand, Vincent Huynh, Ryan G. Wylie, Stephen Boulton, Jinfeng Huang, Chris P. Verschoor, Giuseppe Melacini

**Affiliations:** a Department of Biochemistry and Biomedical Sciences , McMaster University , Hamilton , ON L8S 4M1 , Canada . Email: melacin@mcmaster.ca; b Department of Physics and Astronomy , McMaster University , Hamilton , ON L8S 4M1 , Canada; c Department of Chemistry and Chemical Biology , McMaster University , Hamilton , ON L8S 4M1 , Canada; d Department of Health Research Methods, Evidence, and Impact (HEI) , McMaster University , Hamilton , ON L8S 4M1 , Canada

## Abstract

Soluble amyloid beta assemblies (Aβ_*n*_) are neurotoxic and play a central role in the early phases of the pathogenesis cascade leading to Alzheimer's disease. However, the current knowledge about the molecular determinants of Aβ_*n*_ toxicity is at best scant. Here, we comparatively analyze Aβ_*n*_ prepared in the absence or presence of a catechin library that modulates cellular toxicity. By combining solution NMR with dynamic light scattering, fluorescence spectroscopy, electron microscopy, wide-angle X-ray diffraction and cell viability assays, we identify a cluster of unique molecular signatures that distinguish toxic *vs.* nontoxic Aβ assemblies. These include the exposure of a hydrophobic surface spanning residues 17–28 and the concurrent shielding of the highly charged N-terminus. We show that the combination of these two dichotomous structural transitions promotes the colocalization and insertion of β-sheet rich Aβ_*n*_ into the membrane, compromising membrane integrity. These previously elusive toxic surfaces mapped here provide an unprecedented foundation to establish structure-toxicity relationships of Aβ assemblies.

## Abstract 2

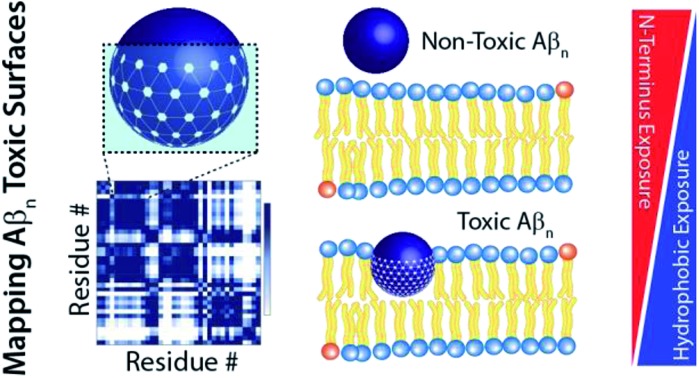
Atomic resolution map of the soluble amyloid beta assembly (Aβ_*n*_) “toxic surfaces” that facilitate the early pathogenic events in Alzheimer's disease (AD).

## Introduction

While the etiology of Alzheimer's disease (AD) is complex and not fully understood, genetic mutations provide compelling evidence that the amyloid beta (Aβ) peptide plays a critical role in AD pathogenesis.^1^,^2^ Indeed, mutations in the genes encoding either the Aβ progenitor (*i.e.* the amyloid precursor protein or APP) or the APP processing enzyme (presenilin 1 and 2 genes) are sufficient to cause AD.^1^ Moreover, none of the familial AD mutations involve genes encoding for the tau protein. Instead, tau mutations enhance the deposition of neurofibrillary tangles *i.e.* the other neuropathological hallmark of AD, but not amyloid plaques, and lead to different neuropathological disorders.^3^ These genetic signatures coupled with the observation that Aβ deposition precedes other biochemical and histopathological changes, including neurofibrillary tangle formation,^4^ provide evidence that tau aggregation occurs downstream to Aβ aggregation. In addition, Aβ clearance is controlled by one of the most significant risk factors for late onset AD, *i.e.* APOE4.^5^ Given the genetic link between Aβ and AD, one of the main hypotheses proposed to explain AD pathogenesis is the amyloid cascade. The amyloid hypothesis posits that neuronal death in AD patients is associated with the increased production, self-association and accumulation of Aβ in the brain.^2^

Since it was originally postulated, the generality of the amyloid cascade hypothesis has been challenged because Aβ plaque burden correlates poorly with cognitive dysfunction.^6^ However, this inconsistency has been reconciled by considering that soluble oligomers and protofibrils formed during the self-association cascade towards mature fibrils are neurotoxic^7^ and better correlate with cognitive impairment in the early stages of AD.^8^ Moreover, the neurotoxicity of Aβ oligomers has been linked to tau hyperphosphorylation,^9^ providing further evidence in support of the upstream role of soluble Aβ assemblies in the AD pathogenesis cascade.^1^

The central role of soluble Aβ oligomers and protofibrils in AD has prompted substantial efforts to identify the molecular determinants of neurotoxicity in soluble Aβ assemblies (Aβ_*n*_, where *n* represents the number of Aβ molecules comprising the assembly).^10^–^22^ Unfortunately, given the transient and heterogeneous nature of Aβ intermediates, characterization of their structure and properties has been challenging. Despite these hurdles, it has been possible to delay the growth of aggregation intermediates to an extent sufficient to enable structural elucidation. For example, Ahmed *et al.* have shown that toxic Aβ_42_ oligomers stabilized through low temperature and salt conditions are largely disordered, but exhibit a turn conformation reminiscent of protofibrils and fibrils.^20^ In contrast, for the other major isoform of Aβ, *i.e.* Aβ_40_, toxic oligomers adopt parallel, in-register β-sheets.^21^ While these studies have provided an initial framework to define structural features of toxic Aβ_*n*_, the location of the “toxic Aβ_*n*_ surfaces” remains unclear. Mapping such surface sites is critical as the exposure of toxic surfaces shared by multiple soluble Aβ_*n*_ species has been hypothesized to be one of the main causes of Aβ_*n*_ toxicity.^1^,^23^

Exposure of these toxic surfaces is thought to facilitate interactions with multiple cellular components, including membranes, which underlie key pathogenic steps in the progression of AD.^1^,^22^,^24^–^26^ In fact, extracellular Aβ oligomers are known to perturb biological and biomimetic membranes at multiple levels. The oligomers can (i) bind to membranes causing local perturbations,^19^,^27^ (ii) form annular structures that insert into the membrane and affect ion homeostasis^16^,^18^,^19^ and (iii) bind to membrane receptors altering signal transduction pathways.^28^ Similar hypotheses have been proposed to explain the neurotoxicity of Aβ protofibrils,^17^ although the latter have been shown to act also through detergent-like permeabilization and eventual fragmentation of the membrane.^19^ While these results highlight critical aspects of Aβ–membrane interactions, the “toxic surfaces” that enable key interactions with the membrane, as well as the underlying mechanism, remain elusive.

As a further step towards dissecting the molecular determinants of soluble Aβ_*n*_ toxicity and mapping the toxic Aβ_*n*_ surfaces, here we systematically investigate a library of Aβ_40_ assemblies sampling different degrees of cellular toxicity. To this end, we first stabilized canonical, toxic Aβ_40_ assemblies through desalting and low temperature^29^ and then treated them with a diverse set of catechins, ranging from (–)-epigallocatechin-3-gallate (EGCG), which remodels Aβ into non-toxic structures,^30^ to (–)-epicatechin (EC), which is expected to detoxify Aβ only partially. We then profiled our soluble Aβ library through multiple complementary techniques with different degrees of spatial resolution, including extrinsic fluorescence, electron microscopy, dynamic light scattering, wide-angle X-ray diffraction and NMR spectroscopy. Unlike previous attempts to dissect the toxicity determinants of Aβ assemblies,^20^,^21^ here we characterize representative soluble Aβ assemblies from our library both in the absence and presence of model membranes.

The comparative analysis of our soluble Aβ_40_ library reveals a cluster of key toxicity determinants and the associated mechanism of action. We discovered that toxicity scales proportionally to the enhanced hydrophobic exposure of Aβ_40_ assemblies and their ability to interact with Aβ monomers and cell membranes. The hydrophobic region spanning residues 17–28 is more accessible to monomer recognition in toxic Aβ_*n*_ relative to Aβ_*n*_ with reduced cellular toxicity. Moreover, whereas increased exposure of hydrophobic residues is required for toxicity, we find that shielding of the highly charged N-terminus, *i.e.* residues < 12, from Aβ monomer recognition enhances the toxicity of Aβ_*n*_. These toxic Aβ_*n*_ surfaces are critical for the binding of Aβ_*n*_ to lipid membranes and for forming membrane-embedded β-sheet structures, which compromise the integrity of the cell membrane. The resulting model provides a foundation to start defining structure-toxicity relationships of Aβ assemblies.

## Results and discussion

### An Aβ_40_ assembly library that samples a cytotoxicity gradient

**Fig. 1:**
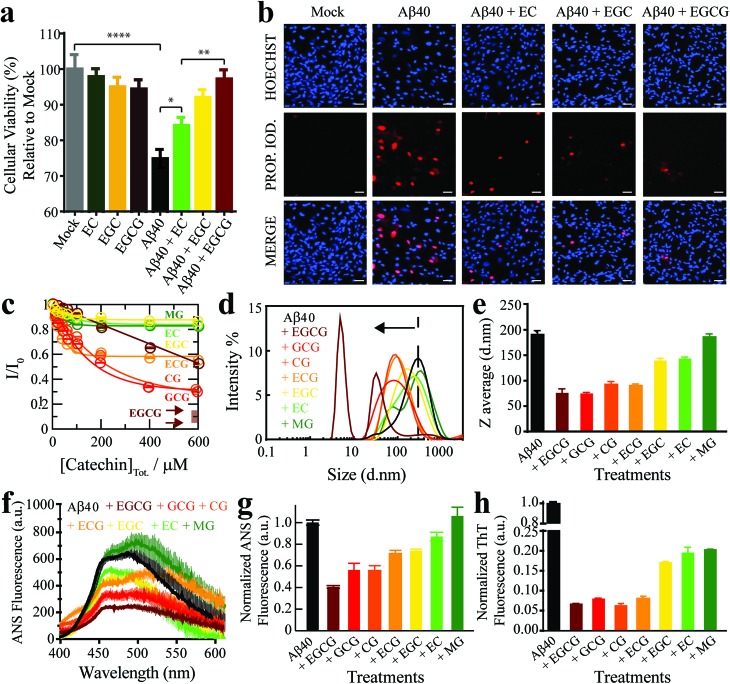
The Aβ_*n*_ library samples a wide-distribution of toxicity, hydrophobic exposure, cross β-sheet content and sizes. (a) Mitochondrial activity of retinal pigment epithelial (RPE1) cells after treatment with representative Aβ_40_ assemblies and associated controls, as monitored by the reduction of resazurin using the PrestoBlue assay.^33^ The data reported show the mean and standard deviation of technical replicates. One-way ANOVA and subsequent Tukey's post-hoc test was used to determine statistical significance between treatments and mock (1X PBS delivery solution), with *, ** and **** representing *p*-values of 0.05, 0.01 and <0.0001, respectively. (b) Representative fluorescence microscopy images of RPE1 cells (scale bar, 50 μm), showing intracellular Hoechst 33342 and propidium iodide fluorescence after incubation with selected Aβ_40_ assemblies. (c) Normalized Aβ_40_ methyl intensity losses upon catechin addition relative to the state in the absence of catechins. (d) Size distribution of Aβ_40_ assemblies in the absence (*black*) and presence of catechins (*coloured as per legend*) as determined by intensity measurements in dynamic light scattering experiments. (e) Z-average of the Aβ_40_ assemblies in (d). (f) ANS fluorescence spectra of Aβ_40_ assemblies in the absence (*black*) and presence of catechins (*colour coded as per the legend*). (g) ANS fluorescence intensities at 454 nm for the samples in (k), normalized to the intensity for Aβ_40_ alone. (h) Thioflavin T fluorescence intensities at 485 nm of Aβ_40_ assemblies in the absence (*black*) and presence of catechins (*coloured as per legend*) normalized to the intensity of canonical assemblies.

As a first step towards dissecting the determinants of Aβ_40_ toxicity, we prepared a library of soluble Aβ_*n*_ spanning a cytotoxicity gradient. For this purpose, we incubated canonical (non-treated) Aβ_*n*_ with a collection of seven distinct catechins expected to remodel to varying extents the pre-existing soluble toxic Aβ_*n*_ into less toxic species^30^–^32^ (ESI Fig. S1,† Methods). Out of this Aβ_*n*_ library, we selected a sub-set of representative Aβ assemblies (*i.e.* those formed in the presence of the EC, (–)-epigallocatechin (EGC) and EGCG catechins) for toxicity profiling in a human retinal pigment epithelial (RPE1) cell line. The state of the RPE1 cells was first monitored by performing PrestoBlue assays, which rely on the reductive potential of the cell as a proxy of cellular viability.^33^ Relative to mock (*i.e.* PBS delivery vehicle), canonical Aβ_*n*_ significantly decrease cellular viability (Fig. 1a, *black vs. grey*). In contrast, Aβ_*n*_ formed in the presence of catechins are less effective in reducing cellular viability, in the order EC (Fig. 1a, *green*), EGC (Fig. 1a, *yellow*) and EGCG (Fig. 1a, *maroon*), for which no significant difference is detected compared to mock (Table S1†). Only negligible changes in cellular viability were observed for cells treated with catechins alone (Fig. 1a, *dark green*, *orange* and *brown*).

We also stained RPE1 cells with the necrotic cell marker propidium iodide (PI), which binds to DNA in cells with severely compromised membranes.^34^ The RPE1 cells were also counterstained with the nuclear marker Hoechst 33342 35 to show that non-specific PI-staining is negligible under our conditions, as indicated by the purple *vs.* red fluorescence for PI in merged *vs.* separate panels, respectively (Fig. 1b). Fluorescence microscopy images of RPE1 cells treated with canonical Aβ_*n*_ indicate prominent staining with PI (Fig. 1b). In contrast, Aβ_*n*_ formed in the presence of catechins exhibit remarkably less PI staining (Fig. 1b), following the same EC < EGC < EGCG ranking as the cellular viability assay (Fig. 1a). Overall, these results suggest that the Aβ assemblies in our library elicit different levels of cellular dysfunction and cell death. Hence, the comparative analysis of such Aβ aggregates is anticipated to reveal key molecular determinants of soluble Aβ toxicity.

### The Aβ assembly library spans a wide distribution of sizes, hydrophobic solvent exposures and cross β-sheet contents

We first evaluated how our catechin library remodels the distribution of Aβ assemblies. For this purpose, the relative populations of the NMR visible low MW Aβ species (*e.g.* monomers) were gauged through residual ^1^H NMR intensities (Fig. 1c), while the NMR invisible Aβ_*n*_ were probed by dynamic light scattering (DLS) (Fig. 1d and e). While it is important to complement these data with size estimations through other means, such as TEM (*vide infra*), interestingly, we observed that all catechins in our library reduce the populations of both the Aβ monomers (Fig. 1c) and the Aβ assemblies at the opposite end of the molecular weight (MW) distribution (Fig. 1d and e). These results suggest that the Aβ species at the extremes of the probability distribution are converted by the catechins into Aβ species with intermediate MW. However, the extent of this remodeling is markedly catechin-dependent with (–)-catechin-3-gallate (CG) leading to large reductions in both the monomer and high MW populations (Fig. 1c–e) and methyl-3,4,5-trihydroxybenzoate (MG) causing only marginal changes (Fig. 1c–e).

We also investigated the surface hydrophobicity of the Aβ assemblies formed under our conditions, as exposed hydrophobic surfaces have been associated with toxicity for another amyloidogenic system.^36^ The surface hydrophobicity of Aβ_*n*_ was probed through 8-anilino-1-naphthalenesulfonic acid (ANS) fluorescence, which exhibits a characteristic blueshift and enhancement in fluorescence intensity upon binding exposed hydrophobic sites. A substantial enhancement in ANS fluorescence was observed for canonical Aβ_*n*_ (Fig. 1f and g, black), whereas the extent of such enhancement is significantly reduced for most catechin-treated Aβ_*n*_ (Fig. 1f and g, coloured). Notably, the measurements of the catechin-treated Aβ_*n*_ surface hydrophobicity (Fig. 1f and g) rank in the same order as the cell toxicities (Fig. 1a), suggesting that exposed hydrophobic surfaces are a key determinant of Aβ_*n*_ toxicity.

Another unique signature of amyloids is the formation of extensive cross β-sheets, as reported by the fluorescent dye Thioflavin T (ThT). Canonical, toxic Aβ_*n*_ exhibit significant ThT fluorescence in comparison to catechin-remodeled Aβ_*n*_ (Fig. 1h). While the decreased ThT fluorescence in the presence of EGCG is in agreement with previous observations,^30^,^37^,^38^ our data on the extended catechin library reveal that other catechins also preserve the ability to destabilize intermolecular β-sheets and/or outcompete ThT. Hence, ThT-responsive β-amyloids do not appear to correlate with cytotoxicity as well as the observables reported above *i.e.* size and hydrophobic exposure. Indeed, solvent accessible hydrophobic moieties are one of the main drivers for Aβ–membrane interactions, which in turn have been proposed as a key determinant of the cytotoxicity associated with Aβ.^39^ This hypothesis is supported by our propidium iodide results, which indicate that toxic Aβ_*n*_ severely compromise the integrity of cell membranes (Fig. 1b). To further corroborate this hypothesis, we evaluated the interactions between a representative subset of our Aβ_*n*_ library and biomimetic membranes (small unilamellar vesicles, SUVs).

### Toxic Aβ assemblies co-localize, bind and insert into biomimetics membranes

**Fig. 2:**
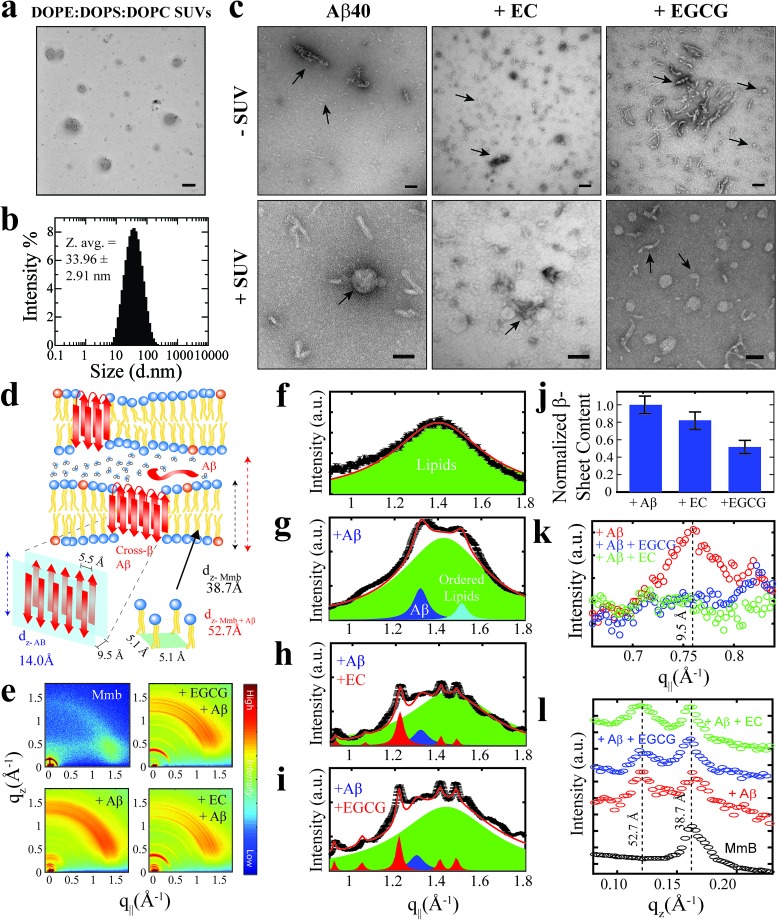
Localization and insertion of Aβ_40_ assemblies into model membranes. (a) Negative-stain TEM image of 800 μM DOPE : DOPS : DOPC SUVs. (b) Size distribution of SUVs shown in (a) as determined through dynamic light scattering intensity measurements. (c) Negative-stain TEM images of Aβ_40_ assemblies in the absence and presence of EC and EGCG and the same assemblies treated with the SUVs in (a) and (b). All scale bars correspond to 100 nm. (d) Schematic summary of the information extracted from wide-angle X-ray diffraction experiments. (e) Complete two-dimensional intensity maps of the X-ray diffraction data with both in-plane and out-of-plane features. (f–i) In-plane (q_‖_) diffraction patterns (*black line*) and fitted Lorentzian peaks (*coloured peaks*) for DOPE : DOPS : DOPC lipids (*green peaks*) in the absence and presence of Aβ_40_ assemblies (*blue peaks*) with and without catechins (*red peaks*). Red lines indicate total fits derived from the summation of component peaks. (j) Normalized population of membrane-embedded β-sheet assemblies relative to canonical Aβ_40_ assemblies, derived through the integration of blue Aβ peaks in (f–i). (k) In-plane (q_‖_) diffraction patterns highlighting the cross-β inter-sheet signal intensity, which correspond to the 9.5 Å spacing between β-sheets shown in (d). (l) Out-of-plane (q_z_) diffraction patterns depicting the membrane lamellar spacing (panel d, dashed black and red lines corresponding to 38.7 and 52.7 Å, respectively) in the absence (*black*) and presence (*coloured as per legend*) of Aβ_40_ assemblies.

We profiled the membrane interactions of selected Aβ assemblies from our library that report on representative regions of our toxicity scale, *i.e.* the canonical as well as the EC- and EGCG-remodeled Aβ_*n*_ (Fig. 1a). For this purpose, SUVs composed of a mixture of DOPE : DOPS : DOPC lipids were prepared with an effective size distribution ranging from ∼10–100 nm and an average diameter of ∼34 nm (Fig. 2a and b). Prior to the addition of the Aβ_*n*_ library to the SUVs, we characterized the morphology of the Aβ_*n*_ by TEM to ensure that significant catechin-induced remodeling occurs. Indeed, compared to canonical Aβ_*n*_, which primarily adopt “worm-like” protofibrils (Fig. 2c, *top left panel*), we observed both spherical assemblies and amorphous aggregates in the presence of EGCG (Fig. 2c, *top right panel*). The latter of the two species has been reported to be an intermediate in the formation of the former.^23^ In contrast, the EC-remodeled Aβ_*n*_ displays features of both canonical and EGCG-remodeled Aβ_*n*_, albeit more closely resembling the canonical Aβ_*n*_ (Fig. 2c, *top center panel*). Having confirmed that catechin-induced remodeling of Aβ_*n*_ occurs, we then evaluated to what extent the Aβ_*n*_ library interacts with SUVs.

TEM images reveal that canonical Aβ_*n*_ significantly colocalize with SUVs. For example, it is possible to observe select Aβ_*n*_ co-positioned with the lipids (Fig. 2c, *bottom left panel*). Similar to the canonical Aβ_*n*_, EC-remodeled Aβ_*n*_ are also somewhat colocalized with the SUVs (Fig. 2c, *bottom center panel*). However, in stark contrast to both the canonical and EC-remodeled Aβ_*n*_, the EGCG-remodeled Aβ_*n*_ are on average spatially distinct from the SUVs (Fig. 2c, *bottom right panel*).

**Fig. 3:**
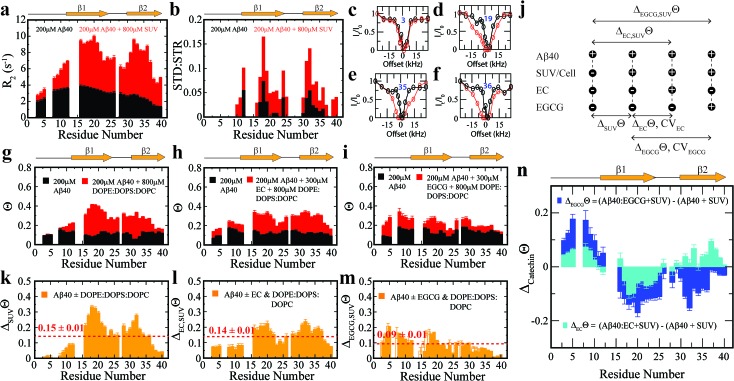
Exchange dynamics of Aβ_40_ monomers on the surface of soluble Aβ_40_ assemblies and model membranes. (a) ^15^N-R_2_ and (b) MeSTDHSQC for the canonical Aβ_40_ assemblies in the absence (*black*) and presence (*red*) of DOPE : DOPS : DOPC SUVs. (c–f) Representative ^15^N-DEST profiles for the samples shown in (a). (g) ^15^N-Θ profiles for the samples shown in (a), colour coding is as per legend. (h) ^15^N-Θ profiles for canonical Aβ_40_ assemblies in the absence (*black*) and presence (*red*) of EC followed by DOPE : DOPS : DOPC SUV addition. (i) ^15^N-Θ profiles for canonical Aβ_40_ assemblies in the absence (*black*) and presence (*red*) of EGCG followed by DOPE : DOPS : DOPC SUV addition. (j) Definition of key differentials in the ^15^N-DEST measurements and the corresponding normalized cellular viabilities. (k) Difference between the Θ profiles shown in (g). The dashed red line indicates the average Θ value. (l) Difference in the Θ profiles shown in (h). (m) Difference between the Θ profiles shown in (i). (n) ^15^N-Θ difference profiles for (h, *red*) *vs.* (g, *red*) (*cyan*) and (i, red) *vs.* (g, *red*) (*blue*).

To complement the TEM data on canonical *vs.* catechin-remodeled Aβ_*n*_–membrane interactions, we performed ^15^N-transverse relaxation (R_2_), ^1^H-based saturation transfer difference (STD) as well as ^15^N-Dark State Exchange Saturation Transfer (DEST) NMR experiments, which collectively probe the interactions of Aβ with high MW (HMW) species, including SUVs, Aβ_*n*_ and their complexes, through the lens of the NMR visible Aβ monomers (Fig. 3a–g).^29^,^40^–^48^ Upon addition of SUVs to the canonical Aβ_*n*_, we observed marked enhancements in R_2_ and STD (Fig. 3a and b), consistent with the Aβ_*n*_–membrane interactions revealed by TEM (Fig. 2c). The SUV-induced changes in R_2_ and ^1^H-based saturation transfer are more pronounced for the residues in the β1 (residues 12–24) and β2 regions (residues 30–40) than for the N-terminal moiety (residues < 12), indicating that the β1 and β2 segments serve as key hot-spots of the SUV-Aβ interactions under our experimental conditions. This conclusion is independently confirmed by the comparative analysis of the ^15^N-DEST data (Fig. 3g–m).

Residues in direct contact with the Aβ_*n*_/SUV surface typically display an attenuation of the residual monomer DEST signal, leading to broadening of the residue-specific ^15^N-DEST *vs.* offset profile relative to amino acids for which the monomer is disengaged from the Aβ_*n*_/SUV surface.^31^ Such broadening of the ^15^N-DEST profile is quantitatively measured through the Θ parameter at intermediate ^15^N-continuous wave (CW) offsets,^40^,^49^,^50^ as explained in the Methods. Consistent with the R_2_ and STD data (Fig. 3a and b), upon SUV addition to canonical Aβ_*n*_, major DEST *vs.* offset profile broadening and corresponding Θ enhancements are observed for the β1 and β2 regions (Fig. 3c–g and k; ESI Fig. S3†). A similar observation applies to the addition of SUVs to EC-remodeled Aβ, which on average display a pattern comparable to canonical Aβ_*n*_ (Fig. 3h and l *vs.*Fig. 3g and k; ESI Fig. S4†). Conversely, the EGCG-remodeled Aβ do not exhibit significant β1 and β2 enhancements as compared to canonical and EC-remodeled Aβ (Fig. 3i and m; ESI Fig. S5†), in excellent agreement with the TEM observations. While the combination of our TEM and ^15^N-based NMR experiments reveal key differences in Aβ–membrane interactions between the less toxic EGCG-remodeled Aβ and the more toxic canonical and EC-remodeled Aβ, they do not provide direct insight about whether Aβ_*n*_ inserts into the membrane and about the structural features of membrane-embedded Aβ_*n*_. To this end, we conducted wide-angle X-ray diffraction (WAXD) experiments in the presence of model membranes for Aβ assemblies at representative regions of our toxicity scale (Fig. 2d–l).

The WAXD two-dimensional intensity maps (Fig. 2e) were modeled with a series of Lorentzian fits (Methods) to derive structural features both in-plane (q_‖_, Fig. 2f–k) and out-of-plane (q_z_, Fig. 2l) of the membrane. For the lipid sample in the absence of Aβ_*n*_, in-plane and out-of-plane Bragg peaks were observed at 1.41 Å^–1^ (Fig. 2f) and 0.17 Å^–1^ (Fig. 2l, *black*), respectively, corresponding to the formation of bilayer stacks with an effective bilayer width of 38.7 Å and a 5.1 Å spacing between individual lipids (Fig. 2d). Addition of canonical Aβ_*n*_ to these lipid bilayers results in additional in-plane features at 1.32 Å^–1^ (Fig. 2g, *blue*) and 0.76 Å^–1^ (Fig. 2k, *red*), indicating the presence of membrane-embedded Aβ_*n*_ adopting laminated β-sheets with 5.5 Å spacing between adjacent β-strands and 9.5 Å between β-sheet layers (Fig. 2d). Interestingly, we observe an additional peak at 1.51 Å^–1^ (Fig. 2g, *cyan*) corresponding to highly ordered lipids likely in the regions interfacing with the embedded Aβ_*n*_. Moreover, an out-of-plane diffraction pattern is observed at ∼0.12 Å^–1^ (Fig. 2l, *red*) consistent with the presence of Aβ not embedded into the bilayer (Fig. 2d).

Compared to canonical Aβ_*n*_, the EC- and EGCG-remodeled Aβ_*n*_ still preserve extended β-sheets in the membrane (Fig. 2h and i, *blue*), although the relative amounts are decreased in the presence of EC and EGCG, in that order (Fig. 2j). In contrast, neither of the catechin-remodelled Aβ_*n*_ exhibit packing of β-sheet layers (Fig. 2k, green and blue), in agreement with our ThT data (Fig. 2h). Overall, these findings suggest that the toxic Aβ_*n*_ formed under our conditions colocalize, interact and insert into lipid membranes wherein they adopt β-sheet structures. To identify the toxic Aβ_*n*_ surfaces that facilitate these multivalent interactions with the membrane, we comparatively examined the ^15^N-DEST differences between canonical, EC- and EGCG-remodeled Aβ_*n*_ in the presence of model membranes (Fig. 3n, ESI Fig. S2†).

### Toxic *vs.* non-toxic Aβ assemblies in the membrane environment exhibit marked differences in Aβ-recognition profiles

To focus on the effects of the catechins, the canonical Aβ_*n*_ DEST profile (ESI Fig. S2b†) was subtracted from the catechin-remodeled Aβ_*n*_ DEST profiles (ESI Fig. S2c and d †). Since all profiles in ESI Fig. S2b–d† were recorded in the presence of SUVs, the resulting DEST differences (Fig. 3n) report primarily on the catechin-induced remodeling of Aβ monomer–Aβ_*n*_ contacts. Specifically, the EGCG-remodeled *vs.* canonical Aβ_*n*_^15^N-Θ profile differences (Δ_EGCG_Θ) show significant decreases in Θ in the two β-strand regions typically observed in Aβ protofibrils (Fig. 3n, *darkblue*). These losses are consistent with the Aβ monomers being less engaged with the Aβ_*n*_ surface at the two β-strand sites in the presence of EGCG. However, the EGCG-induced disengagement detected for the β1 and β2 regions does not extend to the N-terminal segment, for which a significant enhancement in direct contacts is observed (Fig. 3n, *darkblue*). A similar N-terminal Θ DEST enhancement is observed also upon EC addition (Fig. 3n, *lightblue*), albeit with reduced magnitude (Fig. 3n, *light vs. darkblue*). Likewise, in the β1 region the EC-remodeled Aβ_*n*_ show Θ losses with a reduced extent compared to the EGCG-remodeled Aβ_*n*_ (Fig. 3n, *light vs. darkblue*). However, the DEST pattern observed for the N-terminal and β1 regions does not extend to the β2 segment, for which EC and EGCG result in opposite Θ changes (Fig. 3n, *light vs. darkblue*). These findings imply that exposure of the hydrophobic β1-turn region and concomitant shielding of the N-terminus are two key structural transitions intimately linked to toxicity, as these toxic surfaces modulate interactions with the membrane.

### Selection of molecular determinants of Aβ_*n*_ toxicity

**Fig. 4:**
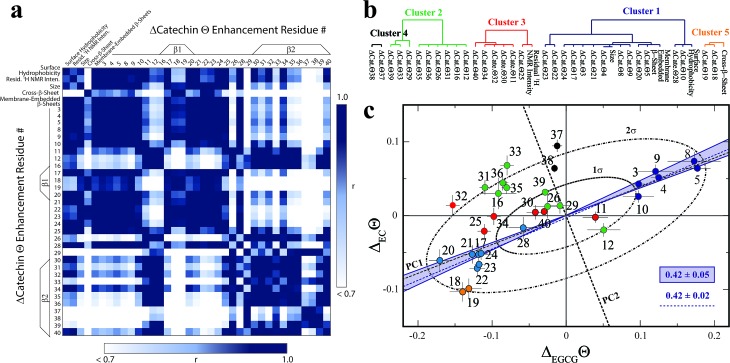
Identification of the determinants of Aβ assembly toxicity through agglomerative clustering and Singular Value Decomposition (SVD). (a) Correlation matrix for the Aβ_*n*_ observables from Fig. 1–3. Correlations with an absolute Pearson's correlation coefficient > 0.95 are indicated in dark blue. (b) Dendrogram displaying the clusters with an absolute Pearson's correlation coefficient > 0.9 obtained through complete linkage agglomerative clustering. (c) Singular Value Decomposition (SVD) of the ^15^N-DEST data. The dashed black lines indicate the first and second principal components (PC1 and PC2) obtained through the SVD of the column-mean centered (Δ_EC_Θ_*i*_, Δ_EGCG_Θ_*i*_) matrix, where *i* is the residue number. The ellipsoids at one and two standard deviations for the residue scores along PC1 and PC2 are shown as black dot-dashed curves. Data for residues assigned to clusters 1, 2 and 3, 4 and 5 though agglomerative clustering are displayed as solid dark/light blue, green, red, black and orange circles, respectively, and the corresponding residue number is reported beside each circle. The solid blue lines define the region of the (Δ_EC_Θ_*i*_, Δ_EGCG_Θ_*i*_) plane that is expected to scale with the relative cellular viability (CV) defined as (CV_Aβ_40_+EC_ – CV_Aβ_40__)/(CV_Aβ_40_+EGCG_ – CV_Aβ_40__) = 0.42 ± 0.05, based on the data of Fig. 1. The dashed blue line (slope of 0.42 ± 0.02 and correlation coefficient of 0.98) was obtained from the linear regression of the DEST data in cluster 1 (blue) and confirms that cluster 1 correlates with cellular viability. PC1 (slope of 0.39) aligns with the residues for cluster 1.

In order to systematically isolate the Aβ_*n*_ features relevant for toxicity, we identified groups of coupled Aβ_*n*_ observables by relying on the data correlation matrix (Fig. 4a), whose elements represent the absolute Pearson's correlation coefficients (|*r*|) between each pair of Aβ_*n*_ observables (ESI†). Through agglomerative clustering of the correlation matrix, we then built a dendrogram that partitions the Aβ_*n*_ observables into five distinct clusters (Fig. 4b). The largest cluster, denoted as cluster 1, includes the Δ_Cat_Θ_*i*_ values for residues in the 3–28 region as well as three low resolution observables, *i.e.* the membrane-embedded β-sheet, the size and the surface hydrophobicity. Since these measurables rank similarly to the relative toxicities (Fig. 1), we hypothesized that cluster 1 defines key molecular determinants of Aβ_*n*_ toxicity. This hypothesis is confirmed by two independent lines of evidence.

First, if we re-compute the correlation matrix and agglomerative clustering after including the relative toxicities (Fig. 1a), we find that the toxicity partitions within cluster 1 (ESI Fig. S6†), confirming that the observables in this cluster scale with Aβ_*n*_ toxicity. Second, in the Δ_EGCG_Θ_*i*_*vs.* Δ_EC_Θ_*i*_ plot (Fig. 4c), the cluster 1 residues fall at or near the region expected to scale with the relative EC *vs.* EGCG cell viability (CV) data, defined as (CV_Aβ_40_+EC_ – CV_Aβ_40__)/(CV_Aβ_40_+EGCG_ – CV_Aβ_40__) = 0.42 ± 0.05 (shaded blue area, Fig. 4c). The linear regression of Δ_EGCG_Θ_*i*_*vs.* Δ_EC_Θ_*i*_ for cluster 1 is in fact in excellent agreement with the value expected based on the relative cellular viability (dashed blue line with slope of 0.42 ± 0.02 and correlation coefficient of 0.98; Fig. 4c). Hence, we conclude that cluster 1 (blue dendrogram in Fig. 4b and blue circles in Fig. 4c) is relevant for the toxicity of Aβ_*n*_.

To gain further insight on the significance of the Δ_EGCG_Θ_*i*_*vs.* Δ_EC_Θ_*i*_ plot and independently corroborate the residue clusters obtained through the agglomerative clustering analysis, we also performed Singular Value Decomposition (SVD) of the data in Fig. 4c. The SVD analysis reveals that the first principal component (dashed black line, Fig. 4c), which accounts for 88% of the total variance, not only resides within the range expected to scale with the relative cellular viability (*i.e*. within the shaded blue area in Fig. 4c), but also aligns with the residues for cluster 1. Interestingly, the SVD reveals that cluster 1 (blue circles, Fig. 4c) is composed of two distinct sub-sets that are mostly confined at opposite extremes of PC1, between the 1σ and 2σ ellipsoids (Fig. 4c). The sub-set with positive PC1 components (dark blue circles) represents the N-terminal residues that become engaged in monomer recognition, as probed by DEST, when cellular viability is enhanced. On the contrary, the cluster 1 sub-set with negative PC1 scores (light blue circles) arises from the β1-turn region residues that become engaged when cellular viability decreases.

In stark contrast to cluster 1, the other clusters obtained from the agglomerative clustering analysis (Fig. 4b, black, green, red and orange circles) fall outside the range expected to scale with cellular viability (blue shaded area, Fig. 4c) and exhibit components along PC2 that are overall higher than those observed for cluster 1 (Fig. 4c). In conclusion, the combined analyses of the correlation matrix, agglomerative clustering and SVD consistently identify the constituents of cluster 1, *i.e*. surface hydrophobicity, size, membrane-embedded β-sheets, N-terminal residue disengagement and β1-turn region engagement, as key molecular determinants of Aβ_*n*_ toxicity.

In order to verify the predictive power of the correlation between Aβ_*n*_ toxicity and cluster 1, we measured the relative toxicities for the Aβ assemblies not included in Fig. 1a and we compared them to those predicted by our model (Fig. 4; ESI Fig. S7†). These Aβ_*n*_ toxicities were not used to train our model and hence provide a critical test of its prognostic capacity. As seen in ESI Fig. S7d,† a strong linear correlation is observed between the predicted and observed toxicities (*r* ≥ 0.94), with a slope within error to one, thus validating the predictive power of our model.

In summary, our investigation of the Aβ_*n*_ library through the comparative analysis of ^15^N-R_2_ and DEST NMR combined with WAXD, TEM, DLS and extrinsic fluorescence reveals key structural differences that distinguish toxic *vs.* non-toxic Aβ assemblies. The integrated analyses of our data through agglomerative clustering and SVD consistently identify a cluster of molecular attributes unique to toxic Aβ_*n*_ (Fig. 4b, cluster 1), including surface hydrophobicity, size, membrane-embedded β-sheets, shielding of the N-terminus and simultaneous exposure of the β1-turn region to Aβ monomers, as probed through DEST NMR.

**Fig. 5:**
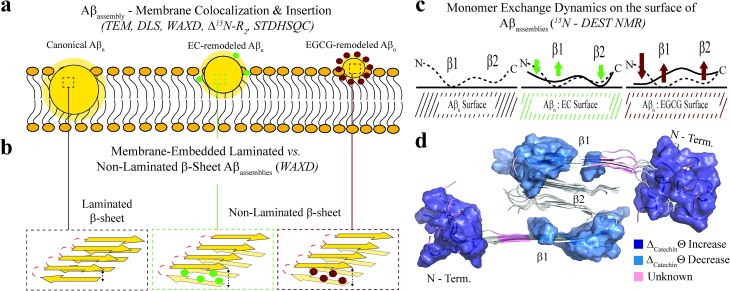
Proposed model for the molecular determinants of Aβ assembly toxicity. (a) Toxic Aβ_*n*_ (canonical Aβ_*n*_) exhibit significant solvent exposure of hydrophobic surfaces (*yellow glow surrounding* Aβ_*n*_). Exposed hydrophobic surfaces facilitate the colocalization, interaction and subsequent insertion of Aβ_*n*_ into the membrane. (b) Membrane-embedded Aβ_*n*_ adopt both laminated and non-laminated β-sheets, indicating that under our experimental conditions the non-laminated β-sheet signature is the minimum structural feature required for membrane insertion and induction of toxicity. (c) Toxic *vs.* non-toxic Aβ_*n*_ exhibit unique regiospecific differences in the recognition of Aβ monomers within a membrane environment. Relative to canonical Aβ_*n*_ (*black*), EC- (*green*) and EGCG-remodeled Aβ_*n*_ (*maroon*) exhibit progressive engagement of contacts with Aβ monomers at the N-terminus and disengagement at the β1-turn region, following the same ranking as their measured toxicities. In contrast, for the β2 region no correlation is observed between toxicity and Aβ_*n*_ monomer recognition. Relevant experimental techniques are indicated in parenthesis. (d) Mapping on the structure of Aβ_40_ fibrils^57^ (PDB code: ; 2LMN) the Aβ residues in cluster 1 (Fig. 4b and c). The N-terminal and β1-turn residues that correlate with toxicity (*blue*) are found in the external regions of the Aβ fibril structure. In contrast, β2 is involved in the lamination of multiple β-sheet layers and is largely inaccessible (Table S2†), explaining its ancillary role in toxicity.

Our data shows that toxic Aβ_*n*_ exhibit solvent exposed hydrophobic sites accessible to ANS binding. While the relationship between surface hydrophobicity and toxicity has been observed previously for several protein systems such as the Type A/B HypF-N assemblies,^51^,^52^ the A^+^/A^–^ Aβ_42_ oligomer pair,^53^ the sup35p oligomer pair,^54^ and others,^55^ here we not only systematically confirm this association for the Aβ system using a library of Aβ assemblies, but we also propose an unprecedented mechanism of Aβ_*n*_ toxicity probed at multiple degrees of resolution. Such mechanism reveals how hydrophobic exposure relates to Aβ–membrane interactions and Aβ monomer recognition. The combination of our TEM, DLS and ^15^N-DEST and R_2_ data collectively shows that Aβ_*n*_ with greater surface hydrophobicity *e.g.* canonical and EC-remodeled Aβ_*n*_ colocalize and interact with the membrane surface more effectively than the less toxic Aβ_*n*_ with less exposed hydrophobic sites *e.g.* the EGCG-remodeled Aβ_*n*_ (Fig. 5a).

The surface hydrophobicity-mediated interactions with the membrane are not limited to the membrane surface, as our WAXD data show that canonical and EC-remodeled Aβ_*n*_ exhibit significant populations of β-sheets embedded in the membrane compared to EGCG-remodeled Aβ_*n*_. The functional effect of the membrane-embedded β-sheets is recapitulated by our propidium iodide-based assay, which indicates that canonical Aβ_*n*_ significantly enhance the permeability of the cell membrane compared to the less toxic Aβ_*n*_ formed in the presence of EGCG.

Notably, we also found that cross-β-sheet structures are dispensable for membrane insertion, as only canonical Aβ_*n*_ exhibit cross lamination of β-sheet layers, whereas EC-remodeled Aβ_*n*,_ with comparable levels of membrane-embedded β-sheets, exhibit considerably reduced cross lamination, similar to EGCG remodeled Aβ_*n*_ (Fig. 2k and 5b). The lack of correlation between toxicity and β-sheet cross-lamination is also consistent with the variability in sheet-to-sheet pairing angles reported for oligomers of model amyloidogenic sequences stabilized by macrocyclic peptides.^56^

The correlation and SVD analyses also identify a cluster of residues confined to the N-terminus and β1-loop region that are key to the regulation of Aβ_*n*_ toxicity (Fig. 4b and c, cluster 1). The probability distribution of contacts between Aβ monomers and the Aβ_*n*_/SUVs surface is markedly enhanced in the β1-loop region (residues 17–28) and concomitantly reduced at the N-terminal segment (residues 3–10) as the Aβ_*n*_ toxicity increases (Fig. 3n and 5c, *green vs. maroon arrows*). Interestingly, an unexpected decorrelation with toxicity is observed at the β2 region (residues 30–40) (*clusters 2 and 4*), for which the EC-remodeled Aβ_*n*_, with intermediate toxicity, exhibits a further enhancement in contacts relative to the canonical Aβ_*n*_ (Fig. 3n and 5c, *green*), in stark contrast to the reduction observed for EGCG-remodeled Aβ_*n*_ (Fig. 3n and 5c, *green vs. maroon arrows*).

Notably, the N-terminus and β1-loop Aβ regions identified by the correlation and SVD analyses to be toxicity determinants (Fig. 4c, cluster 1) are located at the external surface of the Aβ_40_ fibril structure (Fig. 5d, *blue surfaces*). Furthermore, most familial AD mutations (English, Tottori, Iowa, Arctic, Dutch and Italian) that alter the biophysical properties of Aβ are observed in the N-terminal and β1 regions.^1^,^58^ Conversely, the β2 region not identified by SVD as linked to toxicity, is inaccessible to the environment (Table S2†) and is found embedded into the structural core of the fibril, where it is involved in the cross lamination of multiple β-sheet layers (Fig. 5d, *grey cartoon*). These observations agree with our WAXD and ThT data, consistently pointing to β-sheet lamination as accessory to toxicity induction.

## Conclusions

Overall, our data indicate that Aβ_*n*_ toxicity is regulated by the solvent exposure of hydrophobic surfaces, wherein the hydrophobic β1-turn region is more accessible to monomer/SUV recognition, while the highly charged N-terminus is shielded from such recognition. In comparison, the role of β2 appears to be largely ancillary. These toxic surfaces enhance the colocalization, contacts and subsequent insertion of β-sheet rich Aβ_*n*_ into the membrane, leading to compromised membrane stability. Moreover, the proposed model is able to predict relative toxicities solely based on low-resolution measurements, such as size and surface hydrophobicity. Modulation of these properties through small-molecule treatment can be utilized as an effective strategy to reduce the toxicity associated with soluble Aβ assemblies. In addition, soluble oligomers of amyloidogenic peptides with different sequences have been suggested to share a common conformation,^59^ and Aβ is not only relevant for dominantly inherited AD, but also serves as a model system for a broad-range of amyloid disorders. Hence, the cluster of molecular attributes identified here to correlate with toxicity may be transferrable to other amyloidogenic systems.

## Author contributions

R. A. and G. M. designed research; R. A., M. A., A. K., J. B. and V. H. performed research; R. A., A. K., M. R., J. B., R. E., V. H., R. W., S. B., J. H., C. V. and G. M. analyzed data; R. A. and G. M. wrote the paper. All authors have given approval to the final version of the manuscript. This project was funded by the Natural Sciences and Engineering Research Council of Canada (NSERC RGPIN-2019-05990; RGPIN-2018-05585; RGPIN-2016-06450; RGPIN-2014-04514).

## Conflicts of interest

There are no conflicts to declare.

## Supplementary Material

Supplementary information
