# Supplementary material for: Atomic resolution map of the soluble amyloid beta assembly toxic surfaces
Source: Chem Sci. 2019 May 21;10(24):6072–82. doi: 10.1039/c9sc01331h (PMC6585597; doi:10.1039/c9sc01331h)
Supplement: Supplementary file 1 [file SC-010-C9SC01331H-s001.pdf]

Electronic Supplementary Material (ESI) for Chemical Science.
This journal is © The Royal Society of Chemistry 2019

Supplementary Information

for

Atomic Resolution Map of the Soluble Amyloid Beta Assembly Toxic Surfaces

*R. Ahmed[1], M. Akcan[1], A. Khondker[3], M.C. Rheinstädter[3], J.C. Bozelli Jr.[1], R.M. Epand[1], V. Huynh[2], R. G. Wylie[2], S. Boulton[1], J. Huang[2], C. P. Verschoor[4], G. Melacini[1,2]**

Department of Biochemistry and Biomedical Sciences[1], Department of Chemistry and Chemical Biology[2], Department of Physics and Astronomy[3], Department of Health Research Methods, Evidence, and Impact (HEI)[4], McMaster University, Hamilton ON, Canada

*To whom correspondence should be addressed: melacin@mcmaster.ca

SUPPLEMENTARY FIGURES

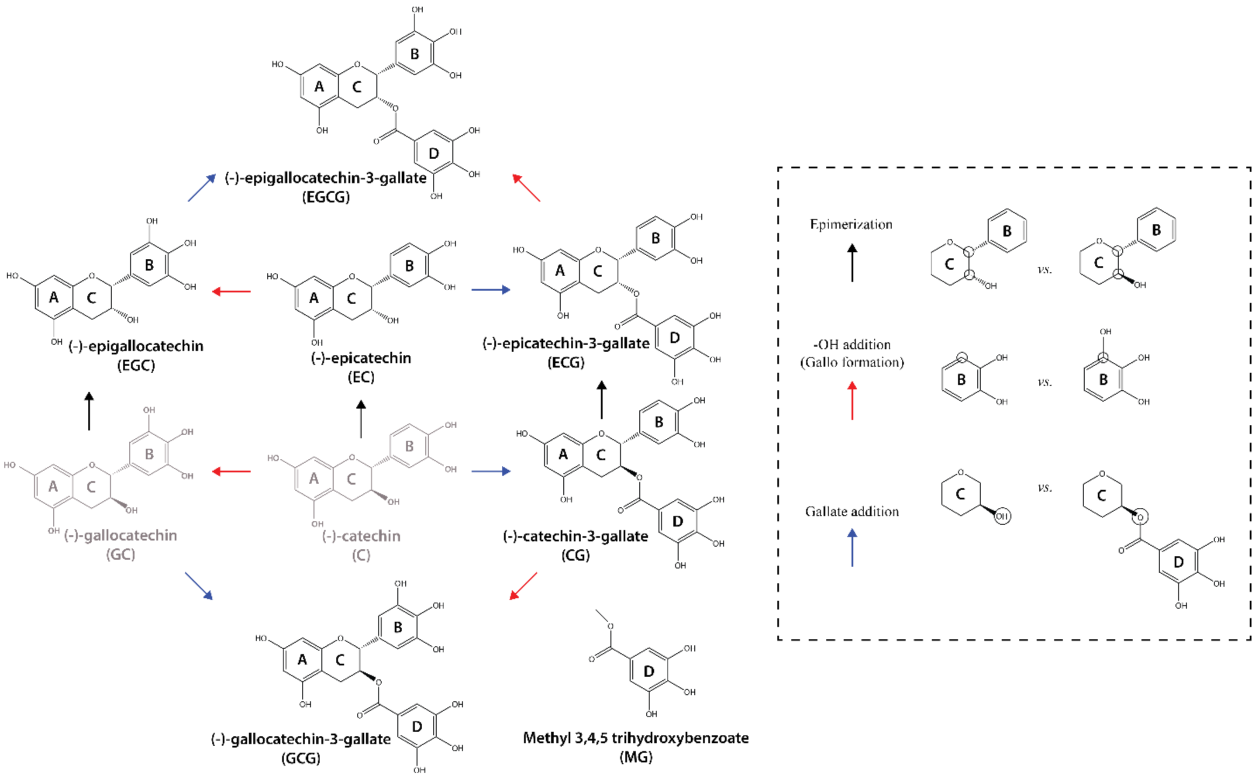

Fig. S1 – Catechin library utilized to modulate Aβ40 toxicity. Arrows represent single substitutions and are categorized as epimerization (*black*), 3'-OH addition to ring B (gallo formation) (*red*), and esterification of the ring C –OH by gallate addition (*blue*). The (-)-catechin and (-)-gallocatechin compounds are shown here for comparative purposes only and were not included in the compound library (*grey*). The arrows are used for illustrative purposes only and do not reflect endogenous synthetic pathways.

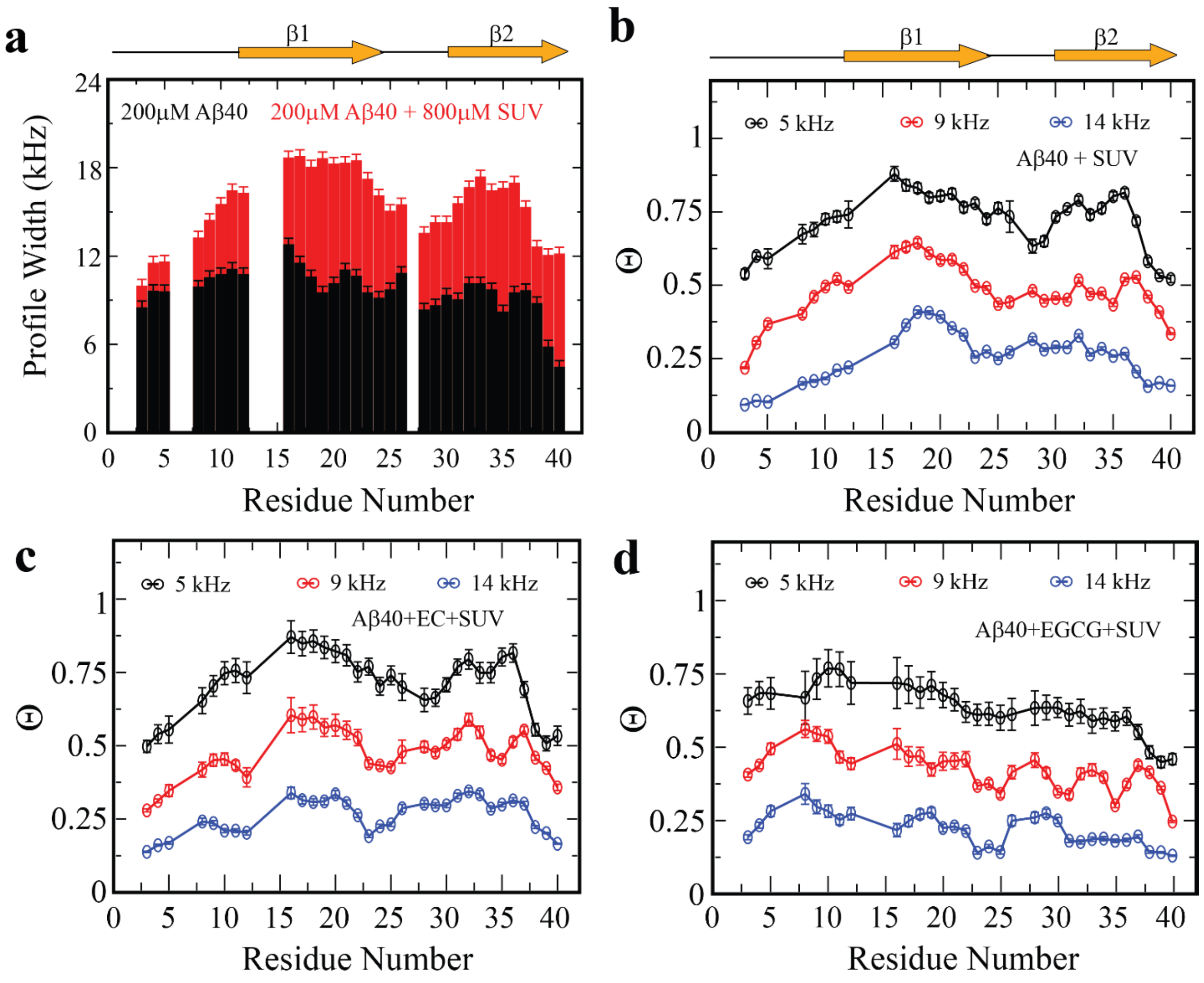

Fig. S2 – Atomic resolution map of the Aβ40$_{monomer}$ exchange dynamics in the presence of DOPE:DOPS:DOPC SUVs. (a) ^{15}N – DEST profile widths for Aβ40 assemblies in the absence (*black*) and presence (*red*) of SUVs. (b) ^{15}N – Θ DEST profiles of canonical Aβ40 assemblies at 5 (*black*), 9 (*red*) and 14 (*blue*) kHz offsets. The ^{15}N – Θ profiles were smoothened by averaging the Θ values for each residue and the two residues directly adjacent to it, when available. (c) As (b) except for the EC-remodeled Aβ40 assemblies in the presence of SUVs. (d) As (c) except for the EGCG-remodeled Aβ$_n$ in the presence of SUVs.

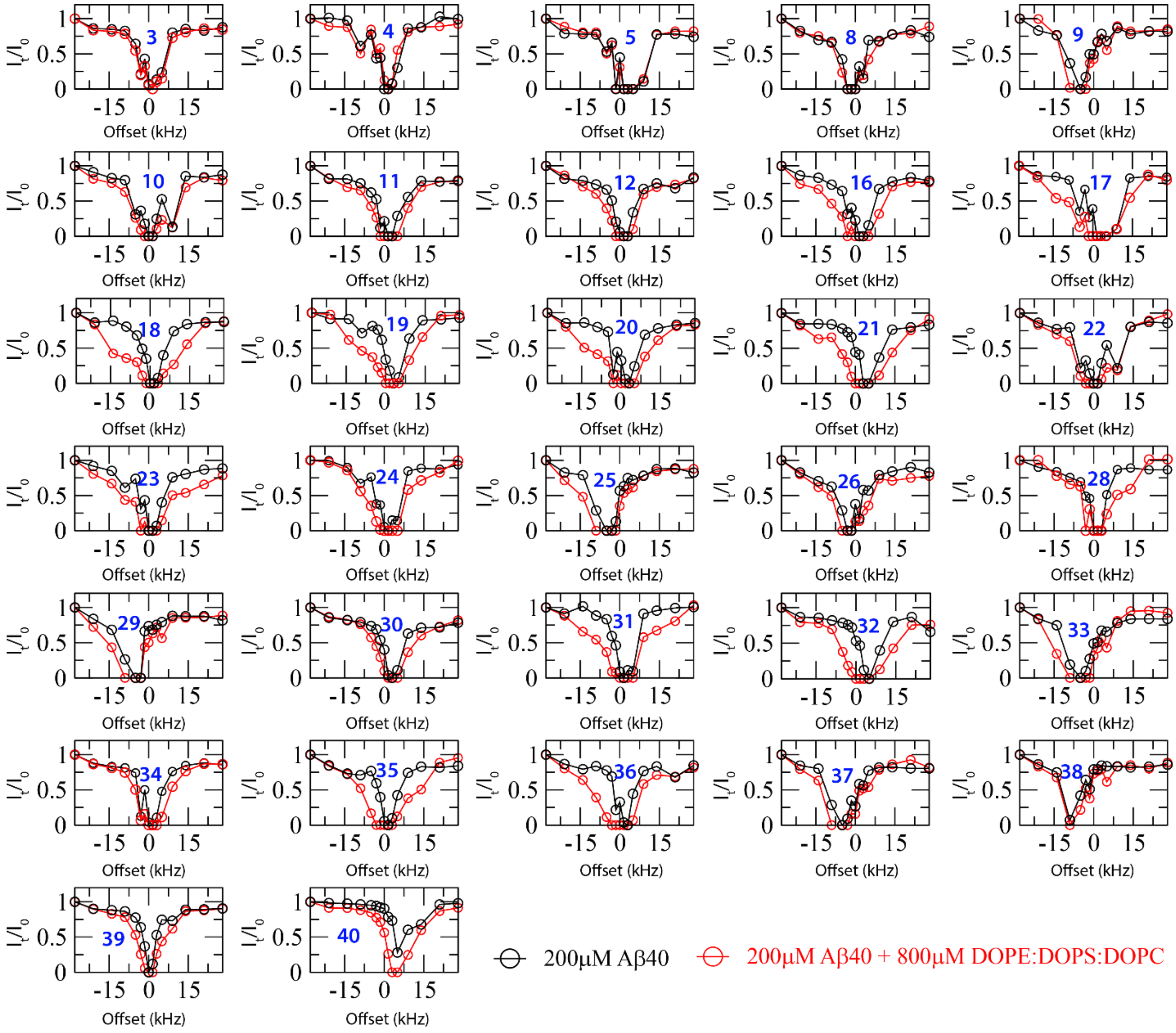

Fig. S3 – Residue-specific ^{15}N – DEST profiles of canonical Aβ40 assemblies in the absence (*black*) and presence (*red*) of DOPE:DOPS:DOPC SUVs. The profiles for residues 3, 19, 35 and 36 from Fig. 3c-f are shown here as well for convenience of comparison.

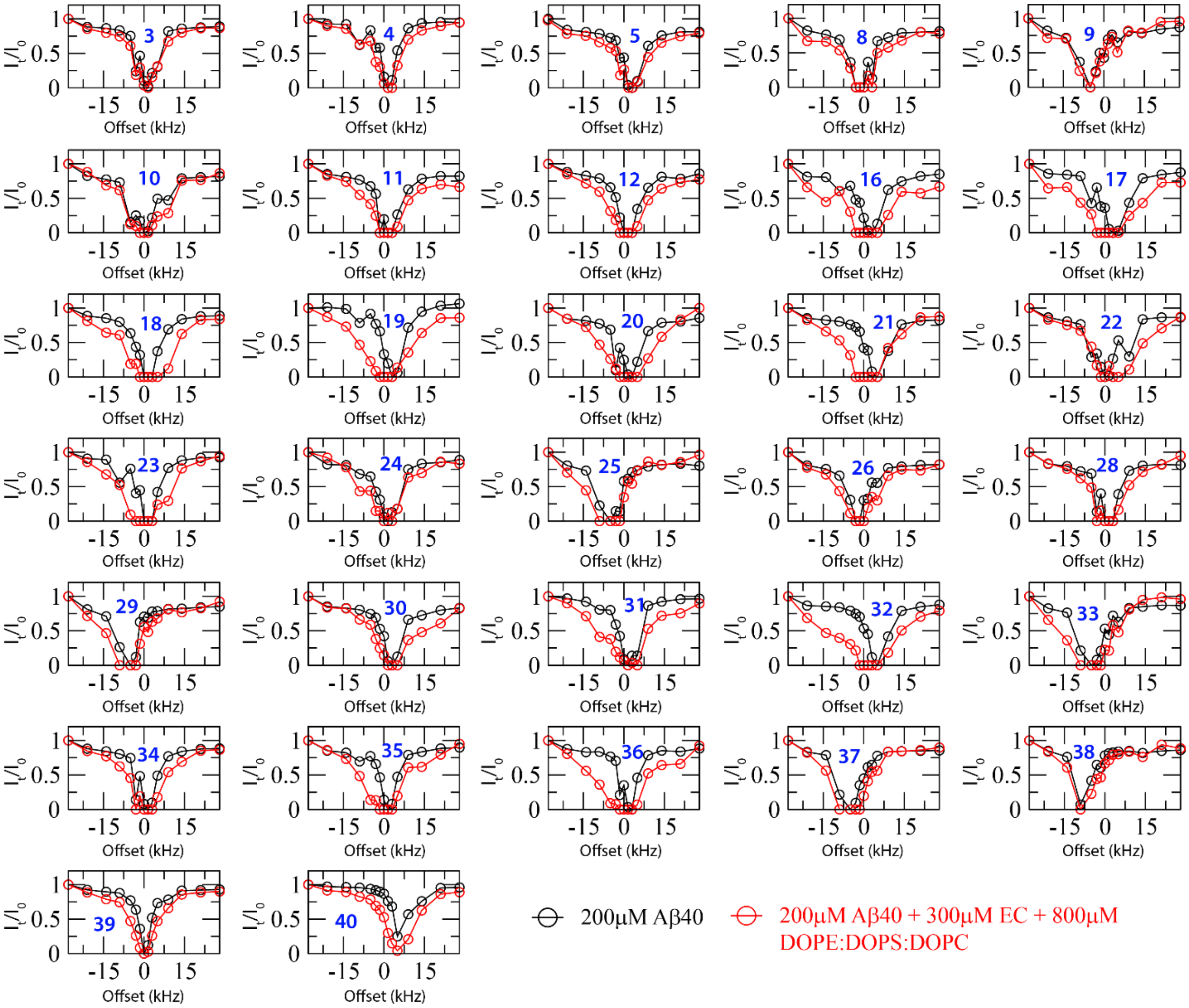

Fig. S4 – Residue-specific ^{15}N – DEST profiles of the ~~EC:~~Aβ40 assemblies in the absence (*black*) and presence (*red*) of DOPE:DOPS:DOPC SUVs.

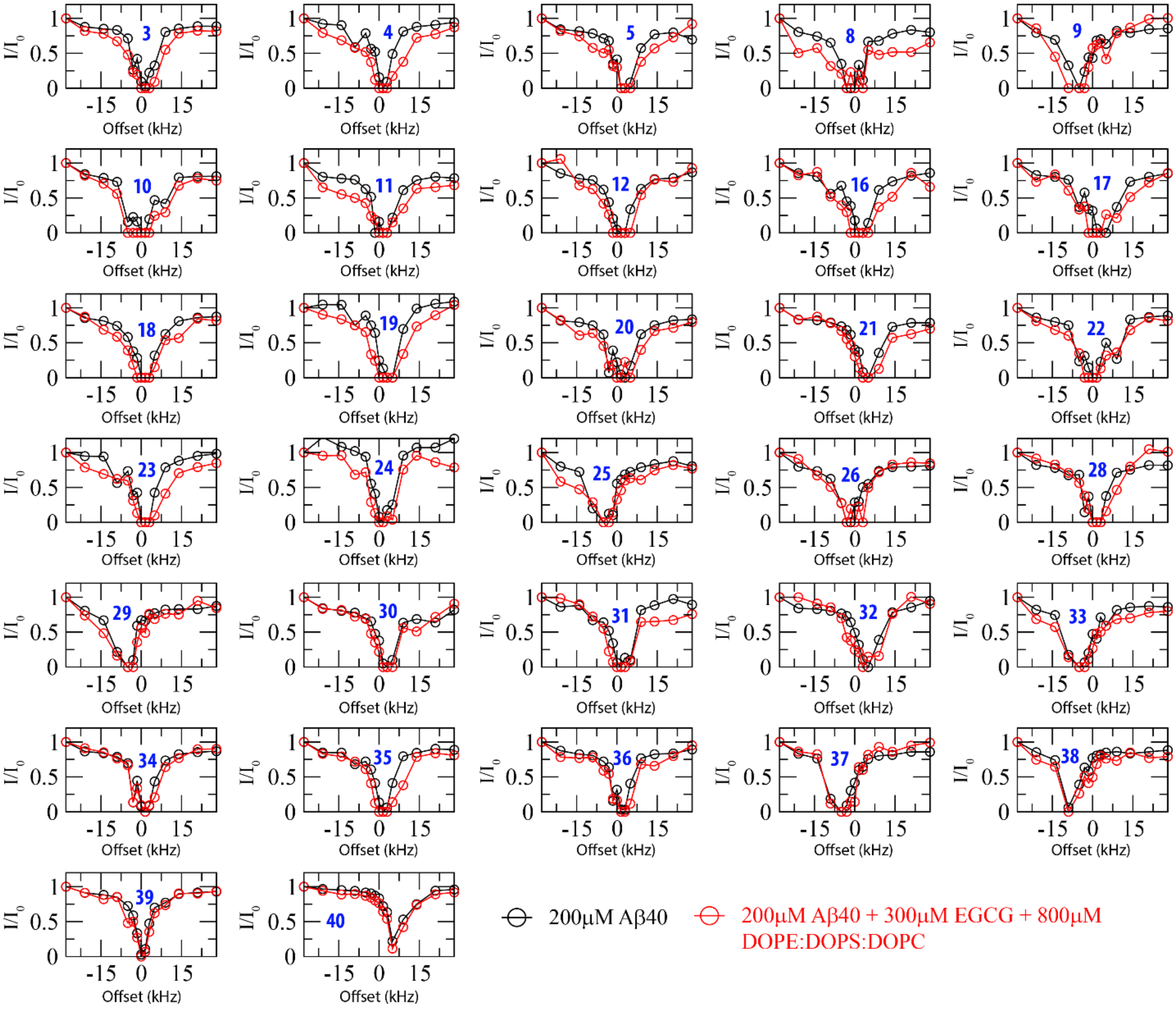

Fig. S5 – Residue-specific ^{15}N – DEST profiles of the ~~EGCG:~~Aβ40 assemblies in the absence (*black*) and presence (*red*) of DOPE:DOPS:DOPC SUVs.

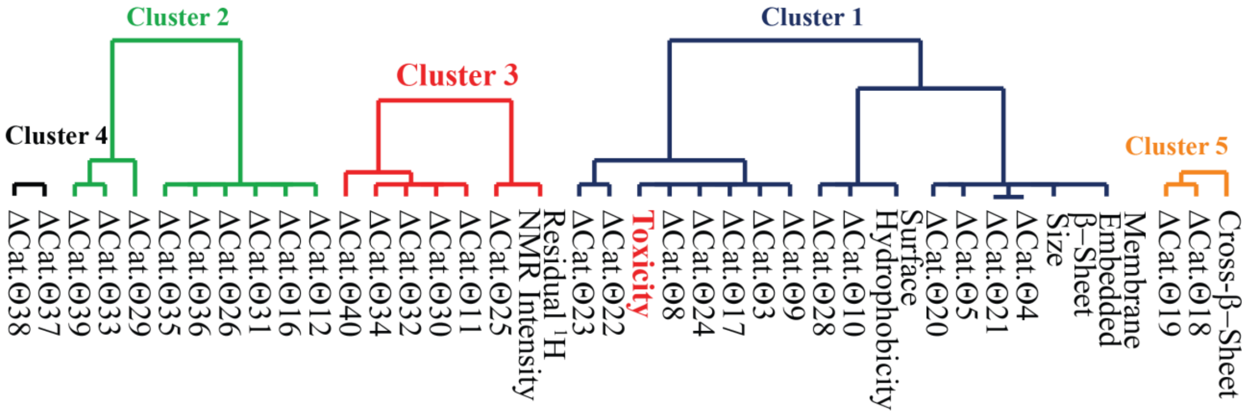

Fig. S6 – Dendrogram displaying the clusters identified through agglomerative clustering of $A\beta_n$ observables, similarly to Fig. 4b, but including cellular viability/toxicity.

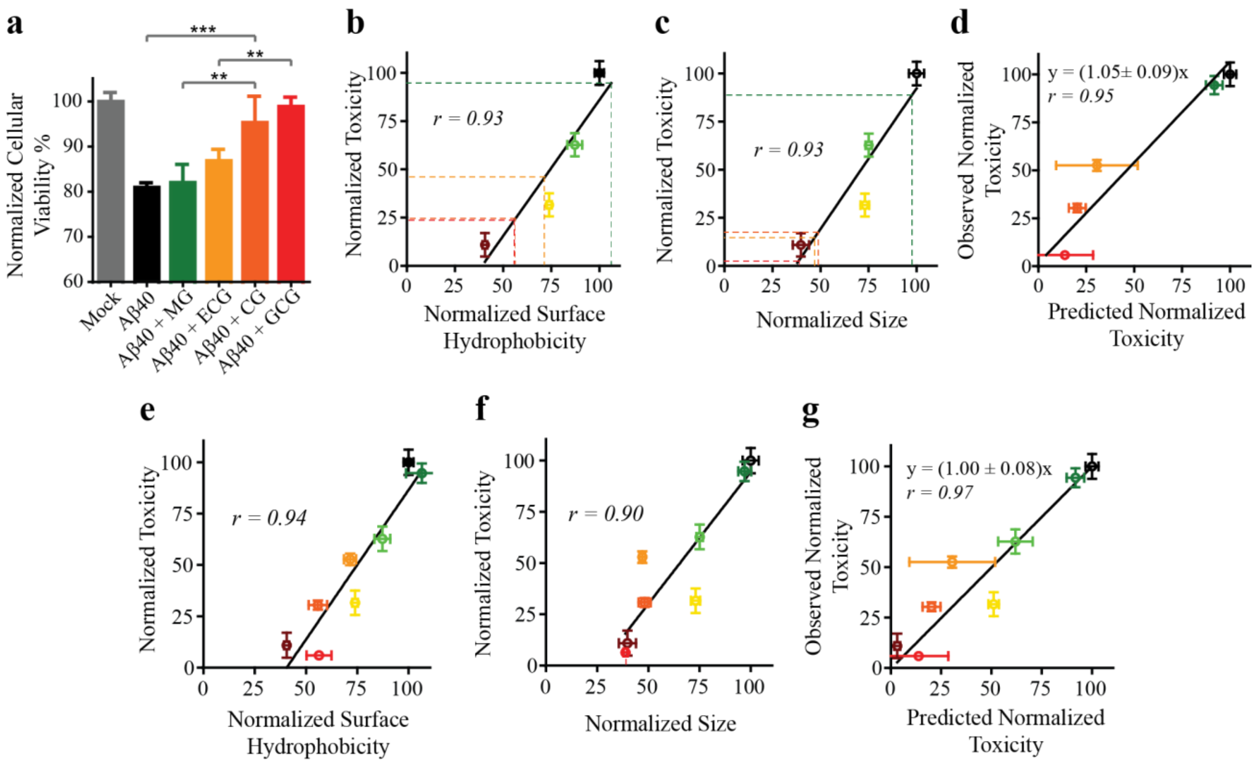

Fig. S7 – Validation of the predictive power of the Aβ_n toxicity model. (a) Cellular viability of the remaining catechin-remodeled Aβ_n not included in Fig. 1a evaluated using the retinal pigment epithelial (RPE1) cell line and the PrestoBlue assay. One-way ANOVA and subsequent Tukey's post-hoc test was used to determine statistical significance between treatments and mock (1X PBS delivery solution), where *, ** and *** represent p values of 0.0423, 0.001 and 0.0005, respectively. The experiments were conducted independently of Fig. 1a using newly prepared Aβ assemblies. (b, c) The toxicities of the Aβ_n evaluated in (a) were predicted using the two cluster 1 observables, size and surface hydrophobicity, for which data is available for all Aβ_n in our library. (b) Relationship between surface hydrophobicity and cellular toxicity (*solid black line*) as determined using the observed toxicities in Fig. 1a for the canonical (*black*), EC- (*light green*), EGC- (*yellow*) and EGCG- (*maroon*) remodeled Aβ_n as inputs. The surface hydrophobicity is normalized to the canonical Aβ_n, which is set to 100. The normalized toxicity is calculated as $(CV_x - CV_{Mock})/(CV_{A\beta} - CV_{Mock})$ where subscript x represents the cellular viability of the Aβ_n for which the normalized toxicity is being calculated. Coloured dashed lines indicate the extrapolation of the Aβ_n normalized toxicities based on their normalized surface hydrophobicity measured in Fig. 1g. (c) As (b) except using normalized size measurements from DLS experiments (Fig. 1e). (d) Relationship between the normalized toxicity predicted from (b) and (c) and the normalized toxicity observed in panel (a). The predicted normalized toxicity for each Aβ_n is an average of the values shown in (b) and (c), and the error is derived from the standard deviation between the two values. The solid black line indicates the linear regression of the data with y-intercept set to zero. (e) Relationship between normalized surface hydrophobicity and normalized toxicity using all Aβ_n in our library as inputs. (f) As (e) except using normalized size. (g) As (d) except using all Aβ_n in our library as inputs.

Table S1 – Pairwise statistical analyses of the RPE1 cellular viability in the absence and presence of the Aβ assembly library.

Species	P-value*
Mock *vs.* EC, EGC and EGCG	0.9947, 0.8692 and 0.8477
Mock *vs.* Aβ40	< 0.0001
Aβ40 *vs.* EC-remodeled Aβ40	0.0478
Aβ40 *vs.* EGC-remodeled Aβ40	0.0003
Aβ40 *vs.* EGCG-remodeled Aβ40	< 0.0001
EC-remodeled Aβ40 *vs.* EGCG-remodeled Aβ40	0.0097
Aβ40 *vs.* GCG-remodeled Aβ40	<0.0001
Aβ40 *vs.* CG-remodeled Aβ40	0.0005
MG-remodeled Aβ40 *vs.* CG-remodeled Aβ40	0.001
ECG-remodeled Aβ40 *vs.* CG-remodeled Aβ40	0.0423
ECG-remodeled Aβ40 *vs.* GCG-remodeled Aβ40	0.0018

*The p-values are generated from the post-hoc Tukey test of the cellular viability data shown in Fig. 1a and Fig. S7a.

Table S2 – Average residue-specific solvent accessible surface area (SASA) ratios for the 2LMN Aβ40 fibril structure

Residue #	Total		Edge		Non-Edge	
	Average SASA Ratio (%)	STDEV	Average SASA Ratio (%)	STDEV	Average SASA Ratio (%)	STDEV
**9**	98.3	4.4	100.0	0.0	97.9	4.7
**10**	83.5	14.4	99.3	0.6	80.3	13.7
**11**	43.0	18.1	66.4	0.7	38.3	15.9
**12**	41.9	11.4	43.0	3.3	41.6	12.5
**13**	44.4	18.0	79.4	13.1	37.4	7.2
**14**	45.5	25.0	66.8	6.9	41.2	25.2
**15**	35.7	20.5	74.8	8.5	27.9	9.8
**16**	51.3	14.8	64.9	6.8	48.6	14.6
**17**	33.6	19.7	55.1	3.8	29.3	18.7
**18**	40.0	15.4	49.2	4.2	38.2	16.3
**19**	25.4	27.9	23.4	11.5	25.8	30.6
**20**	44.5	14.6	62.6	14.4	40.9	12.3
**21**	25.5	19.3	21.0	14.7	26.4	20.6
**22**	38.8	16.0	50.5	24.1	36.5	14.6
**23**	23.8	27.2	14.1	0.1	25.7	29.7
**24**	67.3	24.3	95.1	5.7	61.7	22.7
**25**	71.4	20.9	87.2	4.5	68.3	21.5
**26**	38.5	32.3	38.7	8.6	38.5	35.6
**27**	62.7	30.8	97.7	3.3	55.7	28.9
**28**	38.1	44.8	100.0	0.0	25.8	37.9
**29**	36.0	15.2	46.3	14.7	34.0	15.1
**30**	19.1	12.1	30.4	8.3	16.9	11.7
**31**	18.8	10.7	38.5	3.9	14.9	6.0
**32**	28.5	18.2	63.2	0.9	21.6	9.1
**33**	14.5	9.6	33.8	4.2	10.7	3.3
**34**	20.8	26.9	71.1	8.6	10.8	14.4
**35**	6.2	8.5	22.9	1.3	2.9	3.8
**36**	30.1	22.6	67.9	13.9	22.5	14.8
**37**	9.8	11.8	11.5	4.1	9.5	12.9
**38**	45.4	10.0	49.5	1.1	44.5	10.8
**39**	55.1	16.6	75.8	20.0	51.0	13.3
**40**	96.5	9.2	100.0	0.0	95.8	10.0

The residue-specific solvent accessible surface areas were determined using the GETAREA software provided by the University of Texas Medical Branch.

METHODS

Amyloid Beta Assembly Library Preparation: ^{15}N uniformly labeled Aβ40 was purchased from rPeptide with purity greater than 97%. Similarly, non-labeled Aβ40 was purchased from EZBiolab Inc. with purity greater than 95 %. The commercial Aβ40 lyophilized powders, both labeled and non-labeled, were treated as described previously[50]. Briefly, 1 mg of peptide was temporarily dissolved in 80 µL of 1% NH_4OH/ddH_2O then further diluted to a concentration of 1 mg/mL with ddH_2O. The solution was then lyophilized and resuspended in 10 mM NaOH at 1 mg/mL. The NaOH solution was then divided into aliquots, lyophilized and frozen at -20°C until use. The lyophilized powders were dissolved to form Aβ assemblies through three different methods that serve three distinct purposes, *e.g.* toxicity profiling, low-resolution characterization and high-resolution mapping of Aβ40 assembly-membrane interactions by NMR. Assemblies prepared for cellular viability and membrane permeability assays were created as follows. Non-labeled lyophilized powder was resuspended in 1X Phosphate Buffer Saline (PBS) pH 7.4 with and without catechins to a final concentration of 30 µM Aβ40 and 150 µM catechin. The mixtures were then incubated at 37 °C for 16 hours to form Aβ assemblies. Assemblies prepared for the low-resolution characterization experiments different from cell viability assays were generated by resuspending non-labeled Aβ40 in 20 mM Sodium Phosphate Buffer pH 6.8 with and without catechins. The aqueous buffer was in 100% D_2O for NMR samples, while H_2O was utilized for other techniques. The final concentrations of Aβ40 and catechin were 30 µM and 300µM, respectively. The mixtures were then incubated at 10 °C for 24 hours for assembly formation. Lastly, assemblies for the high-resolution assembly – membrane interaction experiments were prepared as previously described[50]. Briefly, ^{15}N-labeled or non-labeled lyophilized Aβ40 were resuspended in 3 mM Tris pH 8, desalted using a Zeba Column and mixed with 100 mM HEPES pH 6.6 and 20% D_2O at a 1:1 ratio. The final concentration of Aβ40 was ~200µM, as determined by absorbance measurements at 280 nm using an extinction coefficient of 1490 cm^{-1}. The samples were then incubated at 10 °C for seven days until equilibration, as determined through stabilization of Aβ intensities in 2D ^{1}H-^{15}N HSQC spectra. Catechin-induced assemblies were created by the addition of catechins to the pre-incubated samples to a final catechin concentration of 300 µM. The mixtures were then incubated at 10 °C for another 24 hours to allow sufficient remodeling. Although different buffer conditions are required for different sets of experimental techniques, the library of assemblies created within each set was prepared following the same protocol.

Catechin Stock Preparation: (-)-epigallocatechin-3-gallate (EGCG), (-)-gallocatechin-3-gallate (GCG), (-)-catechin-3-gallate (CG), (-)-epicatechin-3-gallate (ECG), (-)-epigallocatechin (EGC), (-)-epicatechin (EC) and Methyl 3,4,5-trihydroxybenzoate (MG) were purchased from Sigma Aldrich, with a purity greater than 95%. A 2.8 mM stock solution was prepared in either 20 mM Sodium Phosphate buffer pH 6.8 and 100% D_2O, 0.05% NaN_3 or 50 mM HEPES, 1.5 mM Tris pH 6.8 and 10% D_2O, as needed to match protein conditions.

Preparation of DOPE:DOPS:DOPC Small Unilamellar Vesicles (SUVs): 1,2-dioleoyl-sn-glycero-3-phosphoethanolamine (DOPE), 1,2-dioleoyl-sn-glycero-3-phospho-L-serine (DOPS), and 1,2-dioleoyl-sn-glycero-3-phosphocholine (DOPC) were purchased from Avanti Polar Lipids. The lipids were stored at -20° C, under Argon. Solutions in chloroform were prepared from the lipids and were mixed to result in a 5:3:2 lipid molar ratio. The lipid mixture was then evaporated under a stream of nitrogen gas and dried thoroughly under vacuum to yield a thin lipid film on the wall of a glass test tube. The thin film was re-hydrated with 50 mM HEPES, 1.5 mM Tris pH 6.8 and 10% D_2O at a concentration of 15 mg/mL and subjected to vortex mixing and sonication until the solution became clear. The concentration of total

phospholipids was confirmed by measuring the amount of inorganic phosphate released after digestion[60].

Cell Culturing: Non-transformed human epithelial cells immortalized with hTERT (RPE1) were originally purchased from A.T.C.C. (Manassas, Virginia). RPE1 cells were cultured in Dulbecco's modified Eagle's medium (DMEM):F-12 and supplemented with 10% FBS and 0.01 mg/mL hygromycin B. Cell cultures were maintained in a 5% CO_2 humidified atmosphere at 37 °C and grown until they reached confluence, up to a maximum of ten passages.

Cellular Viability Probed through Presto Blue Assay: RPE1 cells were seeded at 10,000 cells per well and grown for 24 hours prior to treatments. The cells were then treated with pre-formed Aβ40 assemblies (4 µM final concentration), mock (1X PBS delivery solution) and catechins (20 µM final concentration), and incubated for 48 hours at 37 °C and 5% CO_2. The presto blue reagent (resazurin) was added to each well and the plate was incubated for a further two hours at 37 °C, 5% CO_2. Fluorescence measurements were acquired using excitation and emission wavelengths of 560 and 590 nm, respectively, using a Biotek Cytation 5 plate reader. The error on these measurements was estimated through the standard deviation of five technical replicates.

Cell Membrane Permeability Probed through Propidium Iodide Assay: RPE1 cell membrane permeability measurements were conducted using the same cell growth and treatment protocol as in the cellular viability assays and were implemented in parallel with the cellular viability assay although on a different plate. The membrane diffusible and non-membrane diffusible nuclear dyes Hoechst and propidium iodide were added to wells to final concentrations of 5 µg/mL and 1 µg/mL, respectively. The plate was incubated at 37 °C and 5% CO_2 for two hours and then imaged using a Biotek Cytation 5 plate reader and the DAPI (377,447) and RFP (531,593) channels.

Negative Stain Electron Microscopy (EM): Aliquots of the Aβ40 canonical assemblies devoid of catechins, EC-induced Aβ40 assembly and EGCG-induced Aβ40 assembly samples were taken at the end of each of the ^{15}N-DEST experiments, *i.e.* prior to catechin addition, after catechin addition but prior to SUV addition and finally after SUV addition, to ensure that the EM images are representative of the species probed in the ^{15}N-DEST experiments. The reaction mixtures were diluted 10-folds with ddH2O. Copper EM grids (400-mesh), which had been freshly coated with a continuous layer of amorphous carbon, were glow discharged with 5 mA current for 15 seconds and shortly afterward the grids were floated on 3 µL drops of the diluted assembly reaction mixtures for two 2 min. Excess of sample was blotted with filter paper and the grids were stained with 1 % uranyl acetate for 30 seconds. Grids were loaded in a room temperature holder and introduced into a JEOL 1200-EX electron microscope operated at 80 kV. All images were acquired with an AMT XR-41 Side-Mount Cooled 4 megapixel format CCD camera.

Dynamic Light Scattering (DLS): The samples used for DLS matched those that were imaged by TEM and are therefore also representative of the species probed in the ^{15}N-DEST experiments. DLS measurements were performed using a Zetasizer Nano ZS Instrument (Malvern Instruments, Malvern UK). Autocorrelation functions were accumulated for two minutes at 10 °C with an angle θ of 173° and a 4 mW He-Ne laser operating at a wavelength of 633 nm. All measurements were performed using a 40 µL (ZEN0040) plastic cuvette. The particle diameter detection limit was 0.6 – 6 µm. The viscosity value for water was used in the analysis of all measurements. All the samples were centrifuged for 10 min at 13,000 rpm prior to DLS measurements.

ANS Fluorescence: Aβ40 assemblies prepared as described above were treated with ANS to a final concentration of 200 µM and the mixtures were added to a Corning 96 half-area microwell plate with non-binding surface (NBS) treatment. Fluorescence measurements were implemented using a BioTek Cytation 5 plate reader in spectral scanning mode with excitation at 380 nm and emission reading in the 400-600 nm range. Minimal fluorescence contributions arising from buffer and catechins were subtracted from the respective assembly-containing wells. All measurements were performed with five technical replicates and the standard deviation between replicates was used as an estimation of the error.

Thioflavin T (ThT) Fluorescence: Aβ40 assemblies prepared as described above were treated with ThT to a final concentration of 50 µM and the mixtures were added to a Corning 96 half-area microwell plate with non-binding surface (NBS) treatment. Fluorescence measurements were implemented using a BioTek Cytation 5 plate reader in endpoint mode with excitation and emission wavelengths at 450 nm and 485 nm, respectively. All measurements were performed with five technical replicates and the standard deviation between replicates was used as an estimation of the error.

Wide-Angle X-ray Diffraction (WAXD): The membranes were deposited on single-side 1 × 1 cm^2 polished silicon wafers. To create a hydrophilic surface, the wafers were immersed in Piranha solution (H_2SO_4:H_2O_2, 7/3, vol/vol) for 30 min on a 3D orbital shaker (VWR). The wafers were then washed with ultrapure water (ddH_2O) before membrane deposition. Solutions of lipids, amyloids and catechins were mixed in the same ratios as for the ^{15}N-DEST NMR samples and 100 µL of the solution was applied on each wafer. Samples were slowly dried on an orbital incubating shaker to ensure that the solution spread evenly on the wafer. The resulting membranes were then hydrated in a closed chamber with a saturated salt solution of $Mg(NO_3)_2[6\ H_2O]$ (Sigma) to result in a relative humidity of 50 % over 24 hours at 300 K.

Both out-of-plane (q_z) and in-plane ($q_{||}$) scattering data were obtained using the Biological Large Angle Diffraction Experiment (BLADE) at McMaster University. BLADE uses a 9 kW (45 kV, 200 mA) CuKα rotating anode at a wavelength of 1.5418 Å. Both source and detector were mounted on movable arms such that the membranes stay horizontal during measurements. Focusing, multi-layer optics provided a high intensity collimated 200 µm sized beam with monochromatic X-ray intensities up to 10^8 counts/s. Scattering was detected using a Rigaku HyPix-3000 2D semiconductor detector with an area of 3000 mm^2 and a 100 µm pixel size, as previously described[61]. All scans were carried out at 300 K. The result of such an X-ray experiment is a 2-dimensional intensity map with a large area (0.03 $Å^{-1} < q_z < 1.1\ Å^{-1}$ and 0 $Å^{-1} < q_{||} < 3.1\ Å^{-1}$) in reciprocal space. The corresponding real-space length scales are determined by $d = 2\pi/|Q|$ and cover length scales from about 2.5 to 60 Å, incorporating typical molecular dimensions and distances. To determine the β-sheet signals, the background scattering was fit with an exponential decay. For lipid and protein signals along the in-plane axis, the chain-chain distance was determined from $a_c = 4\pi/(\sqrt{3}\times q_T)$, where q_T is the position of the tail or protein correlation peak. The intensity, $I(q_{||})$, was then modeled with a series of Lorentzian fits incorporating the lipid tail correlation peak, the β-sheet peak[62], and the catechin crystallite peaks[63].

General NMR Spectroscopy: All NMR spectra were recorded at 10 °C using either a Bruker AV 700 spectrometer equipped with a TCI cryo-probe or a Bruker 850 HD spectrometer equipped with a TXI probe. All spectra were analyzed with TopSpin 3.2.1 and Sparky using Gaussian line-fitting. Additional details are discussed below.

Measurement of ^{1}H NMR intensity losses as a function of catechin concentration. Samples of canonical Aβ40 assemblies, prepared as described above, were titrated with increasing concentrations of catechins up to 600 µM. The titration was monitored by ^{1}H NMR spectra acquired immediately after the addition of each catechin aliquot with 128 scans, 32K complex points and a spectral width of 11.98 ppm. Dilution effects upon catechin addition are corrected for. The relative changes in the ^{1}H NMR intensity of the Aβ methyl peaks were modeled according to a single exponential decay model. The plateau intensity was derived through the fitting of the experimental points.

^{15}N Dark-state Exchange Saturation Transfer (DEST) and ^{15}N transverse relaxation NMR. The ^{15}N-DEST experiment was implemented with a 900 ms ^{15}N continuous wave (CW) saturation pulse at 16 different radiofrequency offsets (no saturation, -28, -21, -14, -9, -5, -3, -1.5, 0, 1.5, 3, 5, 9, 14, 21 and 28 KHz) and a field strength of 170 Hz. The experiment was recorded in interleaved mode with 24 scans, 128 dummy scans, a recycle delay of 1.20 s, 200 (t_1) and 2K (t_2) complex points and spectral widths of 14.28 ppm (^{1}H) and 31.82 ppm (^{15}N). All spectral processing was implemented in TopSpin 3.2.1. and transferred to Sparky for peak intensity measurements. The Gaussian line fitting function in Sparky was used to determine the fitted peak heights and the signal-to-noise ratio was used as a measure of error on the fitted peak heights. The DEST difference, denoted here as Θ, was calculated as follows:

$$\Theta = \frac{(I_{28}+I_{-28})-(I_{14}+I_{-14})}{(I_{28}+I_{-28})} \qquad (1)$$

where I_{v} denotes the peak height measured for a given residue at a ^{15}N CW offset of v kHz. [illegible]he ^{15}N transverse relaxation rates were measured using a pseudo-3D pulse sequence with water flip back and sensitivity enhancement. The experiment was recorded with 32 scans, 128 dummy scans, a recycle delay of 1.20 s, 2K complex points for a spectral width of 14.28 ppm in the t_2 dimension and 200 complex points for a spectral width of 31.82 ppm in the t_1 dimension. The total CPMG lengths were 13.88, 27.76, 41.64, 55.52, 69.40, 83.28, 97.16, 124.92, and 152.68 ms. The CPMG pulse train includes ^{15}N spin-echos composed of ^{15}N 180° pulses within two 0.9 ms delays. The pseudo-3D spectra were processed with TopSpin 3.2.1. and the peak height decays were fitted to an exponential in Sparky.

Methyl STDHSQC. Saturation was introduced through methyl irradiation (MeSTD, 50ms Gaussian pulses at a carrier frequency of 417 Hz at 700 MHz) and recorded with a sensitivity enhanced ^{15}N,^{1}H-(HSQC) read out block. Hence, the STDHSQC pulse sequence probes the saturation transferred to the amides of ^{15}N-labeled solutes (*i.e.* Aβ40). The spectra were recorded with 64 scans, 2K (t_2) and 200 (t_1) complex points for spectral widths of 14.1 ppm (^{1}H) and 31.8 ppm (^{15}N), respectively. Reference HSQC spectra were also recorded with the same acquisition parameters, but 16 scans. The spectra were processed on TopSpin 3.2.1 and transferred to Sparky for intensity measurements using the Gaussian fitting function.

Correlation matrix, Agglomerative Clustering and Singular Value Decomposition of Aβ$_n$ Observables:

Correlation matrix. The low-resolution Aβ$_n$ observables *i.e.* surface hydrophobicity, cross-β-sheet content, size, residual ^{1}H NMR intensity and membrane-embedded β-sheet were normalized to canonical Aβ$_n$ and combined with the high-resolution $\Delta_{catechin}\Theta$ data to create a data matrix (**D**). The **D** matrix was organized with the 37 low- and high-resolution Aβ$_n$ observables arranged into rows measured for up to eight Aβ$_n$ states in our library, which were arranged into columns. The absolute Pearson's correlation coefficients ($|r|$) were computed between each set of Aβ$_n$ observables to generate the absolute correlation matrix of **D** transpose. A three-score gradient colouring system was applied to

the correlation matrix, wherein any elements with $|r| \geq 0.95$ was denoted in dark blue, $|r| = 0.85$ in light blue and $|r| \leq 0.70$ in white.

Agglomerative Clustering. Complete-linkage agglomerative clustering based on the correlation matrix of the transposed **D** matrix was performed using the Cluster 3.0 program (http://bonsai.hgc.jp/~mdehoon/software/cluster/) and the dendrogram trees were generated using JavaTreeview (http://jtreeview.sourceforge.net/). Complete linkage is one of the most conservative criteria to assign members to a cluster. The clusters in Fig. 4b were identified using a cut-off value of 0.9 for the absolute Pearson's correlation coefficient.

Singular Value Decomposition. The $\Delta_{EC}\Theta$ and $\Delta_{EGCG}\Theta$ data for all assigned and resolved Aβ40 residues were compiled into a 32 x 2 matrix and column mean centered to result in a new matrix (**M'**). The matrix **M'** was then factorized as $\mathbf{UxSxV^T}$ through Singular Value Decomposition (SVD) through the Octave software (http://www.gnu.org/software/octave/). The slope of the first principal component was computed based on the V matrix as the V_{12}/V_{11} ratio, while the second principal component was assumed perpendicular to the first. The score matrix was computed as **UxS**. The percentage of the total variance accounted for by the first (second) principal component was computed as $S_1^2/(S_2^2+ S_2^2)$ $(S_2^2/(S_2^2+ S_2^2))$.

Computation of the Solvent Accessible Surface Area (SASA) of the Aβ40 Fibril Structure: The SASA was computed through the Getarea software based on the Aβ40 fibril structure (PDB ID: 2LMN)[57]. The missing N-terminal residues were inserted through alignment of the 2M4J structure[64] with residues 9 and 10 of the 2LMN structure. This process was repeated for all twelve protomers in 2LMN. Using AMBER 16[65], the system was then charge neutralized by adding sodium ions and subsequently solvated in a rectangular box of TIP3P water molecules with a minimum solute–wall distance of 12 Å and box dimensions of 124, 112 and 88 Å. The topology and coordinate files were generated using the Amber force field 14SB. Energy minimizations were then carried out prior to gradually increasing the temperature from 0 K to 300 K for a total duration of 100 ps with the first 20 ps in the NVT ensemble and the following 80 ps in the NPT ensemble. Lastly, the system was equilibrated at 300 K with a weak coupling algorithm for 300 ns to generate the final structure. The whole optimization was conducted while restraining the core 2LMN structure *i.e.* the heavy atoms of residues 12-40 for each protomer using a 500 kcal/mol force constant. All simulations were performed on the Shared Hierarchical Academic Research Computing Network (SHARCNET).

SUPPLEMENTARY REFERENCES

38 Lopez del Amo, J. M. *et al.* Structural properties of EGCG-induced, nontoxic Alzheimer's disease Aβ oligomers. *Journal of molecular biology* **421,** 517–524; 10.1016/j.jmb.2012.01.013 (2012).

50 Ahmed, R. *et al.* Molecular Mechanism for the (-)-Epigallocatechin Gallate-Induced Toxic to Nontoxic Remodeling of Aβ Oligomers. *Journal of the American Chemical Society* **139,** 13720–13734; 10.1021/jacs.7b05012 (2017).

57 A. T. Petkova, Y. Ishii, J. J. Balbach, O. N. Antzutkin, R. D. Leapman, F. Delaglio and R. Tycko, PNAS, 2002, 99, 16742–16747.

60 B. N. Ames, *Methods in Enzymology*, 1966, **8**, 115-118.

61 A. Khondker, R. J. Alsop, S. Himbert, J. Tang, A.-C. Shi, A. P. Hitchcock and M. C. Rheinstädter, Scientific reports, 2018, 8, 12367.

62 J. Tang, R. J. Alsop, M. Backholm, H. Dies, A.-C. Shi and M. C. Rheinstädter, Soft matter, 2016, 12, 3165–3176.

63 A. J. Smith, P. Kavuru, K. K. Arora, S. Kesani, J. Tan, M. J. Zaworotko and R. D. Shytle, Molecular pharmaceutics, 2013, 10, 2948–2961.

64 J. Lu, W. Qiang, S. C. Meredith, W. Yau, C. D. Schweiters and R. Tycko, Cell, 2013, **154,** 1257-1268.

65 D.A. Case, R.M. Betz, D.S. Cerutti, T.E. Cheatham, III, T.A. Darden, R.E. Duke, T.J. Giese, H. Gohlke, A.W. Goetz, N. Homeyer, S. Izadi, P. Janowski, J. Kaus, A. Kovalenko, T.S. Lee, S. LeGrand, P. Li, C. Lin, T. Luchko, R. Luo, B. Madej, D. Mermelstein, K.M. Merz, G. Monard, H. Nguyen, H.T. Nguyen, I. Omelyan, A. Onufriev, D.R. Roe, A. Roitberg, C. Sagui, C.L. Simmerling, W.M. Botello-Smith, J. Swails, R.C. Walker, J. Wang, R.M. Wolf, X. Wu, L. Xiao and P.A. Kollman (2016), AMBER 2016, University of California, San Francisco.
